# Rational use of paracetamol among out-patients in a Bhutanese district hospital bordering India: a cross-sectional study

**DOI:** 10.1186/s13104-018-3764-0

**Published:** 2018-09-10

**Authors:** Thinley Dorji, Kinley Gyeltshen, Krit Pongpirul

**Affiliations:** 1 Jigme Dorji Wangchuck National Referral Hospital, Thimphu, Bhutan; 2 Kidu Medical Unit, His Majesty’s Peoples’ Project, Thimphu, Bhutan; 3 Phuentsholing General Hospital, Phuentsholing, Bhutan; 4 Holistic and Oriental Medicine Research Center, Faculty of Medicine, Chulalongkorn University, Bangkok, Thailand; 5 Department of International Health, Johns Hopkins Bloomberg School of Public Health, Baltimore, MD USA

**Keywords:** Paracetamol, Acetaminophen, Health knowledge, attitudes, practice, Self medication

## Abstract

**Objective:**

Paracetamol or acetaminophen is a weak analgesic commonly used worldwide and in Bhutan. It is available across all levels of Bhutan’s health care system and for purchase without prescription. Little is known, however, about patterns of paracetamol use in Bhutan. This study aimed to assess what the Bhutanese population knows about the indications for use of paracetamol, safe use, and common patterns of usage (frequency, dosage). These questions were studied among Bhutanese living in Phuentsholing, a large commercial town at Bhutan-India border.

**Results:**

Among 441 participants, most (72.1%) reported having used paracetamol in the past 1 year. The mean knowledge score was 57.6%; only 30 participants (6.8%) had what was characterized as “good knowledge.” Level of knowledge was positively associated with level of education (p = 0.031). Less than half (41.3%) had a “good attitude” towards use of paracetamol. In practice, few (4.8%) knew the correct dose, including about one in ten who reported exceeding the recommended therapeutic dose. Most knew about side effects (61.2%) and possible allergic reactions (77.3%). Many participants (47.9%) acknowledged that the self-use of paracetamol may not reduce the number of hospital visits.

**Electronic supplementary material:**

The online version of this article (10.1186/s13104-018-3764-0) contains supplementary material, which is available to authorized users.

## Introduction

In many countries, people believe that they need a “pill for every ill” [1]. Analgesics are one of the commonly used drugs worldwide [2]. Paracetamol or acetaminophen is a weak analgesic commonly preferred because of its effectiveness, low prevalence of side effects, and better patient tolerance [3]. In Bhutan, where healthcare is available to all and free of cost [4], paracetamol is recommended and can be obtained without a prescription at all levels of the healthcare delivery system, from primary care outreach clinics to hospital care [5]. Indeed, paracetamol is so common that people in Bhutan’s villages have local names for it, such as *gu*-*men* or *pho*-*leb kar*-*chung* (the white tablet).

Patients and the healthcare system overall benefit from ready access medicines that have proved to be safe and effective [6, 7]. However, self-medication is safe and effective only if users know about the indications, correct use, expected results, and possible side effects associated with any given drug [2, 8, 9]. In Bhutan, anecdotal reports suggest that paracetamol may be both over- and under-used. Though it is commonly recommended by health workers, in many clinical situations patients expect other prescription drugs instead of or in addition to paracetamol. For example, during an outbreak of dengue fever in 2017 [10], patients and relatives did not believe that paracetamol would be sufficient for the control of symptoms [11, 12]. In turn, prescribers question whether patients who are skeptical will comply with recommendations for taking paracetamol. Patients may also be confused or have different expectations for brand-name paracetamol.

The purpose of this study was to assess knowledge, attitude, and practice with respect to the use of paracetamol in Bhutan. This investigation will provide information for understanding patient preferences and compliance in situations where it is indicated.

## Main text

### Methods

#### Study site and participants

A cross-sectional survey was conducted in May 2018 at the outpatient department of the Phuentsholing General Hospital, Bhutan. Phuentsholing is Bhutan’s largest commercial hub located at the Bhutan-India border. Phuentsholing General Hospital caters to 27,658 resident population and a large number of transient populations [13]. The hospital’s outpatient department sees approximately 500 patients/day, the third highest volume of outpatient visits in the country.

Study participants were recruited using a systematic random sampling approach for the selection of patients who visited the outpatient department. In the absence of known prevalence for use, knowledge and attitudes about taking paracetamol, we assumed a 50% probability for any response. We also assumed that about 15% of those who were selected would fail to agree to participate or be unable to complete the interview. To achieve a 5% margin of error, based on a sample selected from a finite population of 700,000, we aimed to survey 441 patients.

Every weekday, 50 lots were selected randomly from a container that contained lots numbered 001 to 441. Every 10 min, the receptionist at the registration counter handed out the lots to Bhutanese patients aged 18 years and older who were invited to participate in this study. Potential participants were assured that agreeing to or declining to participate would not affect their care. Data were collected by three trained enumerators using an interviewer-administered questionnaire (Additional file 1). The interviews were conducted at a separate counter that was used to enhance privacy for the purpose of this study while the patients waited (on an average 30–40 min) to consult a doctor. The enumerators were trained to read and interpret the questions into the local language of the participants (Dzongkha, Sharchokpa, and Lhotshamkha). Basic socio-demographic details such as age, sex, level of education and whether they were a resident or a visitor to Phuentsholing were collected. At the end of the hospital visit, the weight of the patients was measured by the staff at the Lifestyle Related Diseases Clinic using a digital weighing machine to calculate the dose of paracetamol required for body weight [14, 15].

#### Data collection tool

Based on a thorough review of the literature on self-medication generally and paracetamol (indications for use, recommended use, side effects, adverse effects), we designed a questionnaire for the purpose of this study. The contents were reviewed by a team of experts that included a physician, a clinical pharmacologist, and two general doctors. The face validity and construct validity were tested with 20 individuals who were seen for care at the same hospital in the first week of November 2017.

Basic knowledge about the use of paracetamol was assessed using 20 questions. Domains such as the correct indications of paracetamol, dose and frequency, side effects, and possible benefits from self-medicating were assessed. The correct answer to each question was given a score of one out of a total of 20. A score of ≥ 80% was identified as “good knowledge” [16]; a score of ≥ 60–79% as “satisfactory;” and any score < 60% was considered “poor knowledge.”

Patient attitude toward using paracetamol was assessed using 10 questions [17]. Patient’s acceptance of paracetamol as an effective drug when indicated and the influence of friends and family on their perception about paracetamol was assessed. Each question was scored 1 out of 10. A score of ≥ 60% was considered indicative of a “positive attitude;” lower scores were counted as “negative.”

Patterns of paracetamol use were assessed on specific themes such as the prevalence of any self-medication with paracetamol in the past 1 year. Participants were asked to report the reasons they chose to self-medicate, and, if a doctor recommended the use of paracetamol, whether they ever deviated from the recommendations (frequency, dose).

#### Data processing and analysis

The questionnaire was coded and entered into Epi-data Entry version 3.1 (EpiData Association, Odense, Denmark) and analyzed in STATA Version 13 (StataCorp, Stata Statistical Software, licensed Khesar Gyalpo University). The association between patient characteristics (educational attainment, ethnicity, place of residence and categorical characterization of knowledge or attitude were investigated using Chi square tests. The correlation between patient characteristics (age) and knowledge or attitude were investigated using Pearson’s correlation coefficient. Results that occurred with p < 0.05 were considered significant.

### Results

A total of 441 participants were interviewed. The median age of the sample was 28 (IQR 23, 35; range 18–73) years. There were 256 (58.1%) female and 314 (71.2%) residents of Phuentsholing town. Table 1 describes the basic characteristics of the study sample.

**Table 1:** Basic characteristics of the sample studied among patients attending the outpatient department at the Phuentsholing General Hospital, May 2018

Variable	n (%)
Age (years)	
18 to 25	160 (36.3)
26 to 35	178 (40.4)
36 to 45	56 (12.7)
46 to 55	24 (5.4)
56 to 65	15 (3.4)
> 65	8 (1.8)
Sex	
Male	185 (41.9)
Female	256 (58.1)
Education	
Cannot read and write	49 (11.1)
Non-formal education	5 (1.1)
Monastic education	2 (0.5)
Primary school	35 (7.9)
Secondary school	221 (50.1)
Higher education	129 (29.3)
Comorbidities	91 (20.6)
Resident of Phuentsholing	
Resident of town	314 (71.2)
Visitor to this town	127 (28.8)
Ethnicity	
Ngalong	95 (21.5)
Sharchokpa	171 (38.8)
Lhotshampa	115 (26.1)
Others	60 (13.6)

Many of the participants (72.1%) reported treating themselves with paracetamol in the past 1 year. The main reasons given were saving time (68.5%) and cost (35.8%) by avoiding a hospital visit for the conditions they have treated with paracetamol.

**Fig. 1:**
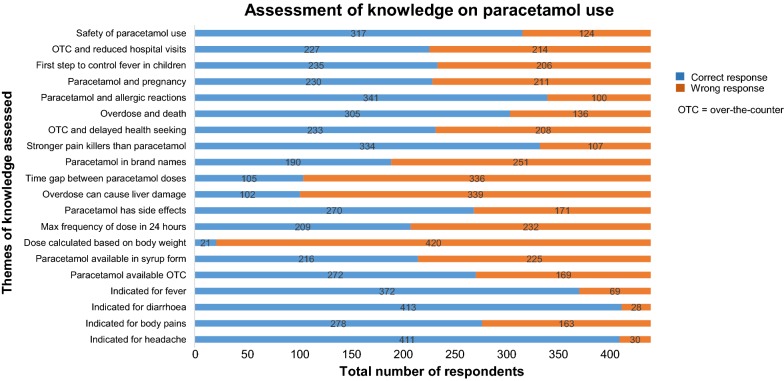
Assessment of the knowledge on paracetamol across various themes among patients attending the outpatient department at the Phuentsholing General Hospital, May 2018

In this sample, just 30 participants (6.8%) had what we would characterize as “good knowledge” about the use of paracetamol (Table 2). The mean score on knowledge was 57.6% (range 25.0–90.0%). The knowledge of the participants under various themes is given in Fig. 1. The level of knowledge of the participants was significantly associated with the level of education (p = 0.031).

**Table 2:** A description of the level of knowledge, the levels of attitude and various aspects of practices on paracetamol use among patients attending the outpatient department at the Phuentsholing General Hospital, May 2018

Variables	n (%)
Levels of knowledge	
Good knowledge (≥ 80%)	30 (6.8)
Satisfactory knowledge (≥ 60 to < 80%)	203 (46.0)
Poor knowledge (≥ 40 to < 60%)	173 (39.2)
Very poor knowledge (0 to < 40%)	35 (8.0)
Levels of attitude	
Good attitude (≥ 60%)	182 (41.3)
Poor attitude (< 60%)	259 (58.7)
Usage of non-toxic dosage	355 (80.5)
Adhere to dose prescribed by doctor	298 (67.6)
Adhere to frequency prescribed by doctor	348 (78.9)
Shared paracetamol with others	293 (66.4)
Storing paracetamol for future use	136 (30.8)
Situations in which paracetamol is used	
Fever	371 (84.1)
Headache	400 (90.7)
Body aches	221 (50.1)
Joint aches	182 (41.3)
Common cold	220 (49.9)
Pain during menstruation	59 (23.0)
Prevalence of self-use of paracetamol	318 (72.1)
Saves time by avoiding visit to hospital	218 (68.5)
Paracetamol is easily available in shops	140 (44.0)
Saves cost by avoiding visit to hospital	114 (35.8)
I can afford to buy paracetamol	85 (26.7)
Source of information	
Doctor	308 (69.8)
Pharmacist	151 (34.2)
Nurse	131 (29.7)
Media	112 (25.4)

Many of the participants (182; 41.3%) had what we characterized as “positive attitude” toward self-medication with paracetamol (Table 2). There was no association between attitude score and the age of participants, ethnicity and the place of residence.

Some participants (47; 10.7%) were found to be using supra-therapeutic dose of paracetamol (19.2–37.5 mg/kg) exceeding a maximum recommended dose of 15 mg/kg [14, 15]. Reported use of paracetamol is shown in Table 2.

### Discussion

This is the first assessment of the use of paracetamol among the adult population in a free healthcare system [4]. While self-medication was prevalent, knowledge about the proper use of paracetamol was very low. Attitude toward the use of paracetamol was mainly negative. Some patients report altering the dose and frequency recommended by the doctor. Taken together, poor knowledge, negative attitudes, and non-compliance will affect whether paracetamol achieves its desired effect. Patients may consider paracetamol less effective than other drugs and request more costly, prescription alternatives when paracetamol would suffice.

Paracetamol is often overlooked both by the physician and the patient in Bhutan. Being an over-the-counter drug, it is generally assumed that the end users have enough knowledge for a rational use of medicine [6, 7].

Other studies in different settings and patient groups have shown that lack of knowledge about proper use of paracetamol may be insufficient to ensure its safe use. Reports from patients attending emergency department [18, 19], those coming to buy over-the-counter paracetamol [20], university students [21, 22] and parents of children [23, 24], recommend the need for educational interventions from physicians and pharmacists. The users need to be given information on the safe use of paracetamol, on the chances of unintentional overdose and on how to calculate the dose of paracetamol and body weight especially in children [23]. They also suggest the need to assess the effectiveness of physician/pharmacist communication [19] in determining patient behaviour that results in the safe use of paracetamol [25].

In our study, the awareness that paracetamol overdose is toxic to the liver was alarmingly low but consistent with findings from other studies [26, 27]. Both accidental overdoses and deliberate self-poisoning are common enough to be of concern [3, 28–31]. Since paracetamol is easily available in many brand names and in combinations, inadvertent overdose is potentially life-threatening and should be considered a significant public health problem [30]. Other studies have shown that youth typically underestimate the potentially lethal effects of paracetamol [3]. Rising rates of deliberate self-harm and suicide in Bhutan [32, 33] make it imperative that the public is aware of the risks of paracetamol overdose.

There are five products that contain paracetamol, alone or in combination with other drugs, in the government’s 2016 National Essential Medicines List for hospitals [5]. However, there are 22 drug companies registered under the Drug Regulatory Authority that supply paracetamol in single or multi-drug formulations to the Bhutanese market through private pharmacies [34]. While the Drug Regulatory Authority regulates the quality and the number of drugs available within Bhutan, the border town of Phuentsholing faces unique challenges. Bhutanese residents can cross the border into Jaigaon, India, and buy any drug without the need to produce a prescription. What follows in border areas and indeed across the country is a potential misuse of drugs for treating conditions that would respond to the proper use of paracetamol. Although paracetamol is available free of cost in Bhutan’s universal health care system [4], patients may prefer and request more costly, prescription-only drugs because of expectations that they will be more effective. Under time pressure during patient encounters [35], Bhutanese physicians may fail to provide the information that patients need for the safe and effective use of paracetamol. Studies in other countries have shown that patients often fail to follow recommendations for proper dose and frequency [29, 36] leading to poor treatment outcomes and poor patient satisfaction [37]. For Bhutan to sustain its free healthcare system in the face of rising health care costs and dwindling donor funds [4, 38], it is imperative to promote the rational use of those drugs in the Essential Medicines List as well as to increase patient satisfaction.

Despite the poor knowledge and attitude towards paracetamol, the self-use of paracetamol was very high because it saved time and cost of a hospital visit. Transportation costs around 26.7% of Nu 7992 (USD 123) as healthcare expenditure for an overnight admission in a hospital in Bhutan [39].

Alteration of paracetamol dose was rampant resulting in the use of supratherapeutic single and cumulative doses. The dosing of paracetamol is recommended at 10–15 mg/kg every 6–8 h and not to exceed a cumulative dose of 4 g in adults and 2 g in children over 24 h [14, 15, 27]. In an event of paracetamol toxicity, therapy in Bhutan is guided by clinical parameters and timely plasma levels of paracetamol tests are not available [40].

### Conclusion

The findings of this study show that while the prevalence of paracetamol use is generally high, the knowledge and attitudes about safe and effective use are generally poor. Clinicians and public health officials should work together to bridge the gap in the rational use of paracetamol.

## Limitations

This study was conducted in one large hospital in one location in Bhutan. The results may not be generalizable to patient experience in other hospitals or clinics across the country. In addition, only those who were actually seeking health care were surveyed; results from a population survey might be considerably different. No information about physician practice was obtained, nor were hospital pharmacists surveyed, to determine how they provide information to patients about safe and effective use, as well as risks. Nevertheless, the results shed light on patient behavior, with information that can be used to increase public awareness about the effectiveness and risks associated with paracetamol.

## Additional file

**Additional file 1.** Knowledge, attitude and practice on paracetamol use among out-patients at the Phuentsholing General Hospital: a cross-sectional study. The pro forma used for data collection in May 2018.
