# Supplementary material for: Rational use of paracetamol among out-patients in a Bhutanese district hospital bordering India: a cross-sectional study
Source: BMC Res Notes. 2018 Sep 10;11:660. doi: 10.1186/s13104-018-3764-0 (PMC6131944; doi:10.1186/s13104-018-3764-0)
Supplement: Supplementary file 1 — Additional file 1. Knowledge, attitude and practice on paracetamol use among out-patients at the Phuentsholing General Hospital: a cross-sectional study. The pro forma used for data collection in May 2018. [file 13104_2018_3764_MOESM1_ESM.docx]

*Data collection pro forma*

*Please 🗸 or fill in the blank wherever instructed. Choose only one response unless otherwise indicated in parenthesis.*

|  | **KNOWLEDGE** |  |
| --- | --- | --- |
| K01 | Paracetamol can be taken for headache. | ⬜ Yes  ⬜ No  ⬜ Don’t know |
| K02 | Paracetamol can be taken for any other body pains. | ⬜ Yes  ⬜ No  ⬜ Don’t know |
| K03 | Paracetamol can be taken for diarrhoea. | ⬜ Yes  ⬜ No  ⬜ Don’t know |
| K04 | Paracetamol can be taken for fever. | ⬜ Yes  ⬜ No  ⬜ Don’t know |
| K05 | Paracetamol can be bought from medical shops even without prescription from a doctor. | ⬜ True  ⬜ False  ⬜ Don’t know |
| K06 | Paracetamol is available in the form of syrup. | ⬜ Yes  ⬜ No  ⬜ Don’t know |
| K07 | When you fall ill, do you know how much of paracetamol you should take for it to be effective? | ⬜ Based on body weight  ⬜ Based on age of the person  ⬜ Based on severity of disease  ⬜ Don’t know how much to take  ⬜ Others, please specify __________________ |
| K08 | Do you know the maximum number of times you can take paracetamol in a day? | ⬜ _________ (number of times)  ⬜ Don’t know |
| K09 | Sometimes, medicines can have effects other than the intended beneficial effects. Does paracetamol also have side effects? | ⬜ Yes  ⬜ No  ⬜ Don’t know |
| K10 | What is the most dangerous thing paracetamol can cause if you take in excess? | ⬜ Eye damage  ⬜ Kidney damage  ⬜ Liver damage  ⬜ Brain damage  ⬜ Don’t know |
| K11 | After you have taken a dose of paracetamol, how many hours will you have to wait for the next dose? | ⬜ _________ (number of hours)  ⬜ Don’t know |
| K12 | Do you know that paracetamol manufactured by different companies are sold under different commercial brand names? | ⬜ Yes  ⬜ No  ⬜ Don’t know |
| K13 | Do you know that there are stronger pain killers other than paracetamol? | ⬜ Yes  ⬜ No  ⬜ Don’t know |
| K14 | Sometimes when you take paracetamol, you feel that you are feeling better. But do you know that this may give you a false image of wellness and delay you in getting the actual diagnosis and treatment? | ⬜ Yes  ⬜ No  ⬜ Don’t know |
| K15 | Paracetamol, if taken in over dose, can cause death. | ⬜ True  ⬜ False  ⬜ Don’t know |

| K16 | Paracetamol can cause severe allergic reaction (skin rash, skin itchiness, shortness of breath, dizziness, palpitations, diarrhoea, watery mouth, etc.) in some persons. | ⬜ True  ⬜ False  ⬜ Don’t know |
| --- | --- | --- |
| K17 | Paracetamol is safe to be given to a pregnant mother (ie, it doesn’t harm the mother and the baby). | ⬜ True  ⬜ False  ⬜ Don’t know |
| K18 | If your child has fever, which step will you take first? | ⬜ Give paracetamol first  ⬜ Tepid sponging with tap water first  ⬜ Don’t know |
| K19 | If you can medicate with paracetamol on your own, do you know that you can cut down the number of hospital visits? | ⬜ Yes  ⬜ No  ⬜ Don’t know |
| K20 | Do you know that your doctors give you paracetamol because it is one of the safest medicines for pains and fever? | ⬜ Yes  ⬜ No  ⬜ Don’t know |
| G09 | Of whatever knowledge you have on paracetamol, from who have you gained this knowledge? ***(Tick all that applies.)*** | ⬜ Doctor  ⬜ Pharmacist  ⬜ Nurses  ⬜ Media  ⬜ Others, please specify __________________ |
|  |  |  |
|  | **ATTITUDE** |  |
| A01 | When a doctor prescribes me paracetamol, I think it is the best medicine for me. | ⬜ Agree  ⬜ Neutral  ⬜ Disagree |
| A02 | The doctor gives me paracetamol because he doesn’t have knowledge about the disease I am having. | ⬜ Agree  ⬜ Neutral  ⬜ Disagree |
| A03 | When I take paracetamol as prescribed by the doctor, it is effective in curing my fever. | ⬜ Agree  ⬜ Neutral  ⬜ Disagree |
| A04 | I think that paracetamol is given unnecessarily to me. | ⬜ Agree  ⬜ Neutral  ⬜ Disagree |
| A05 | Being able to buy paracetamol from the shops will reduce my dependence on doctors. | ⬜ Agree  ⬜ Neutral  ⬜ Disagree |
| A06 | The paracetamol supplied by the government has inferior quality than the ones bought from medical shops. | ⬜ Agree  ⬜ Neutral  ⬜ Disagree |
| A07 | The type of paracetamol that costs more money is better in quality than the others. | ⬜ Agree  ⬜ Neutral  ⬜ Disagree |
| A08 | My friends and family have told me that paracetamol is a good medicine. | ⬜ Yes  ⬜ No |
| A09 | Have you ever asked your doctor why he has given you paracetamol? | ⬜ Yes  ⬜ No |
| A10 | Have you ever requested your doctor to prescribe you paracetamol? | ⬜ Yes  ⬜ No |
|  |  |  |
|  | **PRACTICES** |  |
| P01 | How many tablets of paracetamol do you take at a time when you have headache? | __________ tablets (number) |
| P02 | When you need to take paracetamol, how many times do you take the doses in 24 hours? | __________ times (number) |
| P03 | After your doctor has given you paracetamol, do you increase or decrease the dose of paracetamol on your own? | ⬜ Yes  ⬜ No |
| P04 | Do you follow the frequency (how many times in a day) of paracetamol as advised by your doctor? | ⬜ Yes  ⬜ No |
| P05 | Have you shared your paracetamol with others who have similar illnesses? | ⬜ Yes  ⬜ No |
| P06 | Do you store or keep paracetamol at home for future use? | ⬜ Yes  ⬜ No |
| P07 | For what conditions have you used paracetamol? *Tick all that applies.* | ⬜ Fever  ⬜ Headache  ⬜ Body aches  ⬜ Joint aches  ⬜ Common cold  ⬜ Pain during menstruation  ⬜ Cramps  ⬜ Others, please specify __________________ |
| P08 | In the past year, did you treat yourself with paracetamol without consulting a doctor? | ⬜ Yes  ⬜ No → *If No, Go to Question G01* |
| P09 | Why did you treat yourself with paracetamol without visiting a doctor? ***(Tick all that applies.)*** | ⬜ To save time visit to hospital  ⬜ To save cost of visit to hospital  ⬜ Because I can afford to buy paracetamol  ⬜ Paracetamol is easily available in shops  ⬜ Others, please specify __________________ |
|  |  |  |
|  | **GENERAL QUESTIONS** |  |
| G01 | How old are you? | __________ years (age in numbers) |
| G02 | What is your sex? | ⬜ Male  ⬜ Female |
| G03 | How much do you weigh? | __________ kg (weight in numbers) |
| G04 | What is your ethnicity? | ⬜ Ngalong  ⬜ Sharchokpa  ⬜ Lhotshampa  ⬜ Others, please specify _________________ |
| G05 | Please mention whether you are | ⬜ Resident of Phuentsholing  ⬜ A visitor to Phuentsholing |
| G06 | What is your highest level of education? | ⬜ Cannot read and write  ⬜ Non-formal education  ⬜ Monastic education  ⬜ Class PP to VI  ⬜ Class VII to XII)  ⬜ Higher education (diploma, degree or higher) |
| G07 | Do you have any long term disease? | ⬜ Yes  ⬜ No |
|  |  | -end- |
